# Improving Utility of Data on Cancer Mortality Risk Associated with Smokeless Tobacco: Recommendations for Future Research

**DOI:** 10.31557/APJCP.2019.20.2.581

**Published:** 2019

**Authors:** Rakhi Dandona, Manu Raj Mathur, G Anil Kumar, Lalit Dandona

**Affiliations:** 1 *Public Health Foundation of India, Gurugram, National Capital Region, India,*; 2 *Institute for Health Metrics and Evaluation, University of Washington, Seattle, USA. *

**Keywords:** Smokeless tobacco, chewing tobacco, snuff, cancer mortality, cancer risk, longitudinal studies

## Abstract

**Background:**

We analyzed in detail the studies utilized in most recent global systematic review of risk of cancer mortality with smokeless tobacco (SLT) use to report challenges in the available data that limit the understanding of association between SLT use and cancer mortality.

**Methods:**

For each study, we documented study design, reporting of mortality risk by type of SLT variant, SLT use frequency, and sex of SLT user for oral, oesophageal, pharyngeal, laryngeal and orolaryngeal cancers. These findings are discussed within the context of prevalence of SLT use by geographic regions and sex.

**Results:**

Majority of studies reported mortality risk for oral (70.6%) followed by oesophageal cancer (38.2%). The availability of population-based evidence was low (35.3%). The geographic distribution of studies did not reflect the geographic distribution of countries with high SLT consumption; 61.8% of the studies were from India followed by Sweden (20.6%). Hospital-based (84.2%) studies reported risk with chewing tobacco and the population-based studies (61.5%) with non-chewing tobacco. Hardly any study reported on a particular type of SLT. Definition of SLT use as current, ever or former was limited without consideration of the wide variations in frequency and duration of use within these patterns. Mortality risk reporting for males dominated all cancers other than oral (50% males).

**Conclusions:**

Unless the methodological and generalizability challenges identified in this review are addressed in future research to develop a stronger scientific basis of the association of SLT use and cancer mortality, we would continue to face significant challenges in monitoring the health effects of SLT.

## Introduction

Smokeless tobacco (SLT) consists of a number of products containing tobacco, which are consumed without burning through the mouth or nose (Centre for Disease Control., 2002). The range of SLT products available worldwide is diverse varying in their composition, methods of preparation and consumption (Ministry of Health and Family Welfare GoI., 2017; SEARO. WHO., 2004; Palipudi et al., 2014; Center for Disease Control., 2002). SLT is a complex chemical mixture, which includes components of tobacco leaf, chemicals added during the manufacturing process, the addictive chemical nicotine and more than 20 cancer-causing chemicals including the potent tobacco-specific nitrosamines (International Agency for Research on Cancer, 2008; Stanfill et al., 2011; Richter et al., 2003).

Although, there are a number of biochemical studies showing the presence of carcinogens in various commercially available SLT products, the epidemiological evidence assessing the association between SLT use and cancer mortality is very limited and often not very robust. This lack of strong evidence from major SLT consuming countries has put forward a number of policy challenges seriously affecting efforts to control SLT consumption.

The Siddiqi et al paper, the most recent global systematic review of studies that dealt with risk of cancer mortality with SLT use, reported a significant mortality risk for cancers of mouth, pharynx and oesophagus with any form of SLT use (Siddiqi et al., 2015). Recently, the Global Burden of Disease (GBD) study used more stringent criteria for assessing association of SLT with cancer mortality, which were met by a subset of studies included in the Siddiqi et al systemic review, and reported a significant mortality risk with chewing tobacco only for oral and oesophageal cancers (GBD 2016 Risk Factors Collaborators, 2017). The differences between the two approaches included the different methods of tobacco type mapping, exclusion of hospital-based studies and studies that did not adjust for major confounders in GBD, and calculation of separate relative risks by tobacco type in GBD as compared with pooling of all forms of SLT by Siddiqi et al., (2015); GBD 2016 Risk Factors Collaborators., 2017). Given the varied results produced by the two approaches for association of SLT with cancers, we explored in detail the type of data reported by the individual studies that were identified by the Siddiqi et al systemic review on the association of SLT with cancer mortality to highlight how the lack of standardization of data available in these studies pose challenges in robust assessment of this association. We make specific recommendations for future research that could facilitate availability of data that are more robust to understand association of cancer mortality with SLT use.

## Materials and Methods

*Review of studies*

The main objective of this review was not to conduct a systematic review but to explore in-depth the studies that were included in the most recent global systematic review that dealt with risk of cancer mortality with SLT use. We reviewed each of the studies included in the analysis in the most recent systematic review by Siddiqi et al (Siddiqi et al., 2015). These studies were reviewed in-depth to document the study type, region where it was conducted, type of SLT user for whom the risk was reported, type of SLT variant, and whether the association with cancer mortality was reported for the two sexes separately. As this analysis was based on previously published studies, no ethics approval was necessary. The procedures for review are detailed below.

**Table 1:** Type of Studies Reporting Mortality Risk of Cancers due to Smokeless Tobacco Use that were Documented by Siddiqi et al. 2015.8

Type of cancer	Total number of studies (% of 34)*	Type of study†		
		Population-based	Hospital-based	Mixed
		N (% of total)	N (% of total)	N (% of total)
Any	34	12 (35.3)	19 (55.9)	3 (8.8)
Oral	24 (70.6)	9 (37.5)	12 (50.0)	3 (12.5)
Oesophageal	13 (38.2)	4 (30.8)	7 (53.8)	2 (15.4)
Pharyngeal	7 (20.6)	1 (14.3)	3 (42.9)	3 (42.9)
Laryngeal	6 (17.6)	1 (16.7)	2 (33.3)	3 (50.0)
Oropharyngeal	3 (8.8)	1 (33.3)	2 (66.7)	0

*, Total will add more than 34 as some studies reported on more than one type of cancer; †Population-based studies, cases and controls recruited from population; hospital-based studies, cases and controls recruited from hospitals; mixed studies, either cases or controls recruited form population or hospitals.

**Table 2:** Types of Smokeless Tobacco Use Reported in Various Studies that Reported Association for Cancer Mortality Documented by Siddiqi et al., 2015

Type of cancer/study	Type of smokeless tobacco (SLT) use		
	Chewing tobacco	Other than chewing tobacco	Generic SLT
Oral Cancer (N=24)			
Population-based studies	Tobacco quid	Oral snuff	
	Tobacco chewing	Moist snuff	
	Pan (with or without tobacco)		
Hospital-based studies	Pan (with or without tobacco)	Tobacco snuff	SLT with or without additives
	Khaini, zarda, betel quid with tobacco		
	Tobacco flakes		
	Mishri and supari (crude products)		
	Gutkha, Paan masala		
	Betel leaf (blends and mixed products)		
	Tobacco chewing		
	Betel quid, chewing tobacco		
	Tobacco with or without pan		
	Betal leaf (blends and mixed products)		
	Betel quid with tobacco		
Mixed studies	Tobacco chewing		SLT
Oesophageal Cancer (N=13)			
Population-based studies		Oral snuff	
		Moist snuff	
Hospital-based studies	Tobacco chewing	Snuff	SLT
	Areca nut, betel quid with or without tobacco		
	Gutkha and nass		
Mixed studies	Tobacco quid		
Pharyngeal Cancer (N=7)			
Population-based studies	Tobacco quid		
Hospital-based studies	Tobacco chewing		SLT
	Khaini, zarda, mawa, pan, gutkha		
Mixed studies	Tobacco chewing		
Laryngeal Cancer (N=6)			
Population-based studies		Oral snuff	
Hospital-based studies	Tobacco chewing		
	Khaini, zarda, mawa, pan, gutkha		
	Tobacco chewing		
Mixed studies			SLT
Oropharyngeal Cancer (N=3)			
Population-based studies		Oral snuff	
Hospital-based studies	Tobacco chewing		
	Betel nut with or without tobacco		

**Table 3:** Definitions Smokeless Tobacco Use Reported in Various Studies that Reported Association with Cancer Mortality Documented by Siddiqi et al. 2015

Current use of smokeless tobacco	Ever use of smokeless tobacco	Former use of smokeless tobacco
Daily chewing	Habitual use of chewing tobacco	Chewers who had abstained chewing for at least 12 months before cancer diagnosis or interview
Daily chewing of tobacco quid	Occasional use of chewing tobacco	Habitually chewed it in the past
Daily chewing of tobacco	Habitually used daily for a month	Tobacco chewing in past
Habitually chewed tobacco currently	Daily use of snus	Previous daily consumption of snuff
Tobacco chewing currently	Chewing of tobacco at least once a day for a minimum period of 6 months	Regular former snus use
Chewing of tobacco per day	Ever use of snus	Previous use per day
Daily chewing of tobacco	Regularly use of oral snuff per week	Stopped using tobacco at least 6 months before the interview
Habitually chewed at the time of the interview	Ever use per day	Stopped habitually use of snus at least 1 year before the diagnosis
Habitual daily chewers	Use of moist snuff ever in life time	Stopped using quid or snuff 2 or more years before the interview
Regular current user	Use of snuff and chewing tobacco	
Use oral snuff 1 year prior to the time of interview	Ever use of smokeless tobacco	
Current use per day	Chewed tobacco products at least once a week for a minimum of 6 months	
Present daily use	Chewed or practiced snuff dipping at least once a day for minimum 1 year	
Stopped using moist snuff within the year before diagnosis of cancer	Ever use weekly for a period of 6 months or more	
Current use of smokeless tobacco	Chewed tobacco in their life time	
Use of quid or snuff at least once a week at the time of interview	Use of quid or snuff at least once a week for 6 months or more.	
Currently use of any smokeless tobacco	Use of smokeless tobacco at the time of entry into the cohort	
	Use of tobacco per week for more than one year	

**Figure 1:**
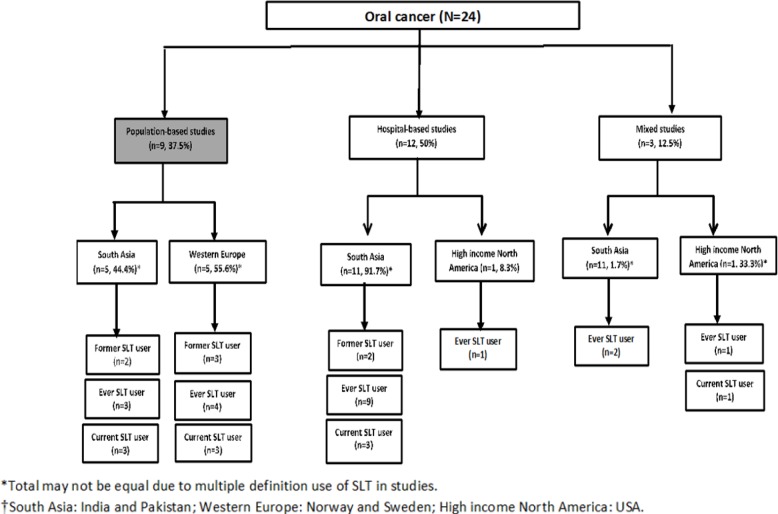
Distribution of Oral Cancer Mortality Risk Studies Based on Type and Location of Study, and Type of Smokeless Tobacco (SLT) User in Studies Documented by Siddiqi et al. 2015

**Figure 2:**
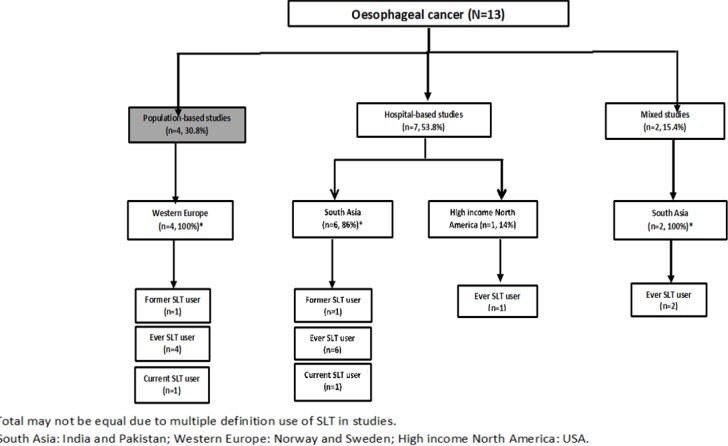
Distribution of Oesophageal Cancer Mortality Risk Studies based on Type and Location of Study, and Type of Smokeless Tobacco (SLT) User in Studies Documented by Siddiqi et al. 2015

**Figure 3:**
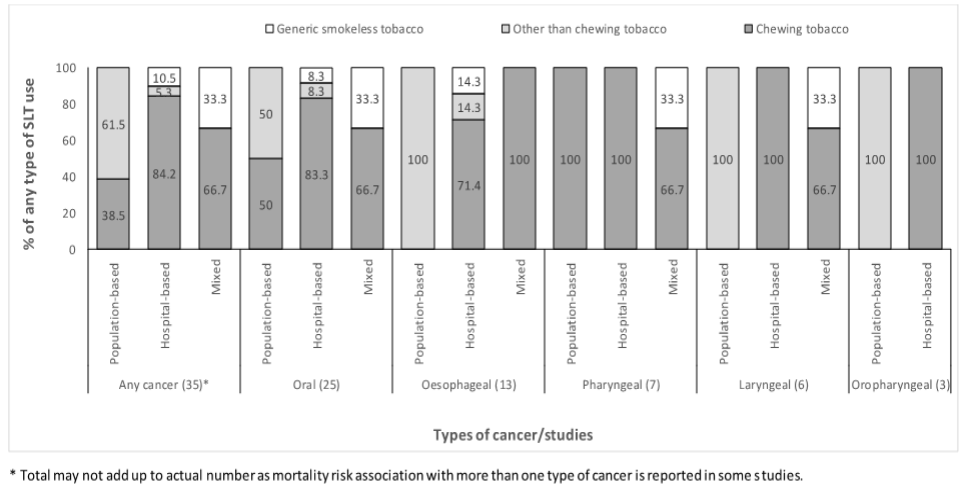
Distribution of Types of Studies Reporting Association for Cancer Mortality by Type of Smokeless Tobacco Use in Studies Documented by Siddiqi et al., (2015)

**Figure 4:**
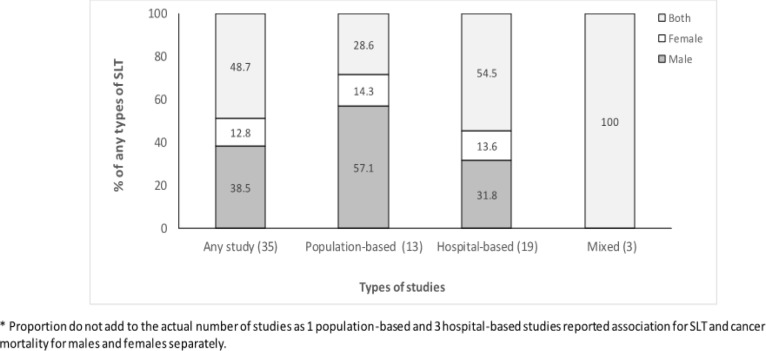
Distribution of Studies Reporting Association for Any Cancer Mortality by Sex for Smokeless Tobacco Use in Studies Documented by Siddiqi et al., (2015).

First, each study was classified into one of the three types based on from where the population for the study was recruited. A study where both the cases and controls were recruited from population was classified as population-based study. Studies with both cases and controls recruited from hospitals were classified as hospital-based, and studies in which cases or controls were recruited both from population and hospitals was classified as mixed studies. Second, each study was then grouped under a region based on where the study was conducted. We used the World Health Organization (WHO) classification for geographical regions of the world. Then, we classified the studies based on the type of SLT user for which the risk was reported. These included – current, ever, and former SLT user. We documented in detail the definitions for each type of SLT user as reported in the studies. Furthermore, the type of SLT variant(s) for which risk association was reported were documented. As there was a significant variety in the type of SLT reported, for a meaningful interpretation we categorized these as chewing tobacco, other than chewing tobacco and generic SLT when no details were available. Lastly, each study was categorized based on whether the risk association was reported for males, females or both. “Both” included the studies wherein no gender-specific risk association was reported. If a study reported risk association for both sexes combined and also for male and female separately, it was included under male and female categories and not both. 

*Analysis*

The results are presented across four themes – by type of cancer, type of SLT variant, type of SLT user, and by sex. Availability of mortality risk data is documented for five types of cancers – oral, oesophageal, pharyngeal, laryngeal and orolaryngeal. If a study reported on risk for more than one type of cancer, then it was counted separately for each type of cancer. Flowcharts are used to describe the variation in availability of data for each type of cancer based on the type of study, the region and type of SLT user. Also, the type of SLT variant assessed for each type of cancer and by the type of study is also presented. Computation of the varieties in type of SLT variant and definition of type of SLT user are presented. Lastly, the sex distribution of the reported mortality risk is presented by the type of study. All analysis was done using MS-Excel.

## Results

A total of 34 studies were considered for association between SLT use and cancer mortality (Table 1) of which, 24 (70.6%) studies reported on association with oral cancer followed by that with oesophageal cancer (n=13; 38.2%). A little over half of the reported studies were hospital-based (n=19; 55.9 %). Twenty-one (61.8%) of the studies were from India, 7 (20.6%) from Sweden, 3 (8.8%) from USA, 2 (5.9%) from Pakistan and one (2.9%) from Norway.

*By types of cancer*

*Oral cancer*

Fifty percent of the studies that reported SLT use association with oral cancer mortality were hospital-based studies (50%) followed by population-based studies (Figure 1). Overall, there was a predominance of studies from the South Asia region (mostly India) and all but one hospital-based study was reported from high income North American (USA) region. Among the 9 population-based studies, a little over half were from Western Europe. Overall, the association for oral cancer was reported for ever or current use of SLT (n=30, 55.6%). In the South Asian region, population-based studies mostly reported association of oral cancer for those currently using SLT (n=3) while most of the hospital-based studies in this region reported association for those who had ever used SLT (n=9).

*Oesophageal cancer*

Figure 2 shows the distribution of studies reporting association with oesophageal cancer mortality based on type and location of study, and type of SLT user. A little over half of the studies were hospital-based (n=7, 53.8%) followed by population-based studies (n=4, 30.8%). All the population-based studies were from the Western European region (Norway and Sweden) and reported association of oral cancer for those who ever used SLT (n=4, 100%). The South Asian region (India and Pakistan) were the pre-dominant location for hospital-based studies (n=6, 86%), and all these studies also reported association for those who ever used SLT (n=6, 100%). 

*Pharyngeal cancer*

Only 7 studies in total reported pharyngeal cancer mortality risk. A similar number of hospital-based (n=3, 43%) and mixed (n=3, 43 %) studies and only one population-based study was reported. All three hospital-based studies were reported from the South Asian region (India) and one mixed study (33.3%) was reported from the high income North American region (USA). All hospital-based studies presented association for ever SLT use and the only population-based study presented it for both former and current user of SLT with pharyngeal cancer. 

*Laryngeal cancer*

A total of 6 studies reported on laryngeal cancer mortality risk, mixed (n=3, 50%) studies followed by hospital-based studies (n=2, 33.3%) predominantly. Four of the 6 studies were reported from the South Asia region (66.7%). The only one population-based study was conducted in the Western European region (Sweden). The association for laryngeal cancer was reported with ever SLT use for 5 (83.3%) of the 6 studies.

*Oropharyngeal cancer*

Two (66.7%) of the 3 studies on oropharyngeal cancer mortality risk were hospital based and reported from the South Asia region. Both these studies reported association for ever SLT use and oropharyngeal cancer. Only 1 population-based study was reported from Western Europe.

*By type of SLT variant*

The various types of SLT for which the mortality risk associations were assessed/reported in the studies were categorized as chewing tobacco, other than chewing tobacco and generic smokeless tobacco. The distribution of these broad categories of type of SLT for which the associations were reported with various cancer mortality are shown in Figure 3. Overall, the distribution of reported associations with chewing tobacco, other than chewing tobacco and generic smokeless tobacco was 65.7%, 25.7% and 8.6%, respectively. Considering any type of cancer, non-chewing forms of SLT dominated the population-based studies (61.5%) whereas other chewing tobacco dominated the hospital-based (84.2%) and mixed studies (66.7%). A varied pattern was seen for the type of SLT association reported for the specific types of cancers as shown in Figure 3.

The specific types of SLT use for which the various studies reported associations with cancer mortality is shown in Table 2. The considerable range in what was considered as SLT across the studies is quite evident from the list in Table 2. It is important to note that hardly any study reporting on “chewing tobacco” specifically mentioned a particular product, with most of these being reported as chewing tobacco and some even without tobacco were included. Specificity was seen only for snuff.

*By type of SLT user*

Table 3 highlights the variety in how the current user, ever user and former user of SLT are specified in the studies that reported on associations of SLT with cancer mortality. There were considerable variations seen in both the frequency and duration of use within each type of SLT user. Frequency of SLT use ranged from no specification to at least once a week for a minimum of 6 months; and that for duration ranged from no specification to 2 or more years before the interview to ever.

*By sex*

Figure 4 shows the sex wise distribution of studies based on the type of study for any cancer mortality. Overall, a total of 19 (48.7 %) studies reported associations for SLT with any cancer mortality for both sexes followed by that for male (n=15, 38.5 %). The population-based studies predominantly reported on males (n=8, 57.1 %) and both sexes accounted for all mixed studies. Figure 4 shows the sex wise distribution reported in the population-based studies only. An association for SLT use with cancer mortality for females was reported only for oral cancer (18.2%). Males dominated all cancers other than oral cancer where 50% of the studies were reported for males and 40% for both sexes.

## Discussion

With little over half of the evidence for risk of cancer mortality with SLT use based on hospital-based studies with varied types of SLT and its use, this review highlights the significant challenges that have to be addressed in future studies to facilitate understanding of the true risk of cancer mortality with SLT use.

The first challenge is considering SLT products as a homogenous group without accounting for the diversity in SLT products. The magnitude of cancer mortality risks directly associated with SLT use appears to differ across countries and regions, likely due in part to differences between SLT products which have varying levels of carcinogens and nicotine across different regions and its patterns of use (National Cancer Institute and Centers for Disease Control and Prevention, 2014; International Agency for Research on Cancer, 2008). Also, some SLT products contain other plant materials in addition to varying levels of carcinogens such as areca nut which has carcinogenic properties. Comprehensive risk assessments of SLT use must address complex mixtures of ingredients and their carcinogenic potential. (National Cancer Institute and Centers for Disease Control and Prevention, 2014) Therefore, considering SLT as a homogenous product for assessing its association with cancer mortality or any health effect is not ideal.(Siddiqi K et al., 2015; GBD, 2016; Risk Factors Collaborators, 2017; Critchley et al., 2003) This review clearly highlights that even within the South Asian region with most SLT consumption,(Stepanov et al., 2005.; Siddiqi et al., 2013) the types of SLT products considered in the studies are so varied that it limits a reasonable quantification of the cancer mortality risk by type of SLT product. The risk associated with snuff, on the other hand, is more generalizable as compared with the other SLT products due to homogeneity of products available and mode of consumption. To address this gap, it would be useful for new research studies to proactively consider certain types of SLT products with a very high prevalence rate in populations, and also provide disaggregated data by the different types of SLT products instead of an overarching category such as “SLT” or “chewing tobacco”.

The second challenge is the limited definition of patterns of SLT use as current, ever or former without consideration of the wide variations within these patterns. This review highlights that the epidemiologic studies of SLT use have either less or very varied information about what levels of use are associated with cancer mortality risk. The quantity, frequency and duration of SLT use appeared arbitrary in the reported studies. Again, given these wide variations in what is considered current or ever or former use, understanding of the true risk of cancer mortality with SLT use is further compromised. Furthermore, establishing a dose- and duration- response association of SLT use with various health outcomes requires a uniform definition of SLT consumption. To address this gap, it would be useful for new research/studies to consider standardized definitions of SLT use that are now in place through the WHO’s Global Tobacco Surveillance System (Global Adult Tobacco Survey Collaborative Group, 2011). 

The third challenge is not reporting/considering the epidemiology of SLT use by sex. Most of the studies reported mortality risk only for males, and quite a few others for both the sexes combined. Among youth and adults, males generally show a higher prevalence of SLT use than females (Siddiqi et al.,2015.; GBD, 2016; Risk Factors Collaborators, 2017; National Cancer Institute and Centers for Disease Control and Prevention, 2014; Giovino et al., 2012; Sinha et al., 2012). However, among adults, SLT use by females is similar to or greater than use by males in some countries (GBD, 2016; Risk Factors Collaborators, 2017; Sinha et al., 2012; Giovino et al., 2012; Sinha et al., 2014). This review highlights that even in the South Asian region where SLT use in females is significant and in most countries, higher than males, only 4 of the 23 studies reported cancer mortality risk separately for females. Furthermore, with the patterns of SLT use and attributable cancer mortality by sex also reported to be different (GBD, 2016; Risk Factors Collaborators, 2017; Siddiqi et al., 2013; Giovino et al., 2012; Palipudi et al., 2012), it is imperative that future research/studies consider reporting the risks separately by sex.

The fourth challenge is the less availability of population-based studies to provide the evidence for cancer mortality risks with SLT use. Majority of the studies in this review were hospital-based studies. It also appears that not all studies, especially population-based, were designed as targeted studies specifically to assess the effect of SLT use on various cancer outcomes given the wide variations in definition of SLT and patterns of use. Furthermore, the confounders for which the reported risk ratio in various studies is adjusted for varies in the numbers and type of confounders ranging from tobacco smoking, alcohol drinking, age, socioeconomic status, income, education, occupation, religion, residence, body mass index, diet pattern and genotype (Siddiqi et al., 2015; Sinha et al., 2016) To address this gap, longitudinal studies specifically designed to provide the causality between SLT use and cancer risks should be considered to firmly establish it as a causal factor of various forms of cancer by addressing the relevant confounders. This will be an important step in accurately determining the risk of cancer associated with SLT.

The fifth challenge is geographic limitation in evidence from several countries which have a high prevalence of SLT use. Three quarters of the world’s SLT users live in five countries, India, Bangladesh, Pakistan, the United States, and Myanmar (World Health Organization, 2017; Giovino et al., 2012). Countries like Sri Lanka, Papua New Guinea, Sweden, Afghanistan, Bhutan, Nepal, and Madagascar also have a high SLT consumption (GBD, 2016; Risk Factors Collaborators, 2017; World Health Organization, 2017). This geographic representation is not reflected in the geographic distribution of the studies that have assessed association of SLT with cancer mortality. In this review, the majority of the studies were from India. Furthermore, varying levels of relative risk for cancer mortality by geographic region have been reported previously (Siddiqi et al., 2015; Sinha et al., 2014; Sinha et al., 2016). As already highlighted above, this review suggests that even within the geographic regions with most SLT use, the current evidence does not account for variety of SLT products. For example, in India the most common forms of SLT used are khaini, tobacco with lime, gutka, and betel quid (Ministry of Health and Family Welfare GoI, 2017). In this review, only 5 of the 21 studies from India actually mentioned these SLT products specifically. Therefore, evidence is needed from countries other than India for global understanding of the cancer mortality risk with SLT use, in addition to better understanding based on type of SLT product within the countries with highest consumption.

In conclusion, this review suggests that a wide range of challenges remain in relation to understanding the effect of SLT use on cancer mortality. The data available do not allow for precise quantification of the cancer mortality risk and to identify the SLT related factors that drive the risk. The findings of this review may also be relevant for other health conditions that are reported to be associated with SLT use such as Ischemic Heart Disease (Vidyasagaranet al., 2016). Unless the recommendations made in this paper for the identified methodological and generalizability challenges are addressed in future research to develop a stronger scientific basis of the association of SLT use and different types of cancer mortality, we would continue to face significant challenges in monitoring the health effects of SLT. Some of the recommendations made may also be relevant for the global tobacco surveillance to bring uniformity to the data collected globally (Global Adult Tobacco Survey Collaborative Group, 2011). 

## Funding Statement

This research did not receive any specific grant from funding agencies in the public, commercial, or not-for-profit sectors.
